# Targeting Autophagy in the Tumor Microenvironment: New Challenges and Opportunities for Regulating Tumor Immunity

**DOI:** 10.3389/fimmu.2018.00887

**Published:** 2018-04-25

**Authors:** Bassam Janji, Guy Berchem, Salem Chouaib

**Affiliations:** ^1^Laboratory of Experimental Cancer Research, Department of Oncology, Luxembourg Institute of Health, Luxembourg City, Luxembourg; ^2^Centre Hospitalier du Luxembourg, Department of Hemato-Oncology, Luxembourg City, Luxembourg; ^3^INSERM U1186, Gustave Roussy Cancer Center, Villejuif, France; ^4^Thumbay Research Institute for Precision Medicine – Gulf Medical University, Ajman, United Arab Emirates

**Keywords:** autophagy, hypoxia, tumor microenvironment, immune response, tumor immunity

## Abstract

Cancer cells evolve in the tumor microenvironment, which is now well established as an integral part of the tumor and a determinant player in cancer cell adaptation and resistance to anti-cancer therapies. Despite the remarkable and fairly rapid progress over the past two decades regarding our understanding of the role of the tumor microenvironment in cancer development, its precise contribution to cancer resistance is still fragmented. This is mainly related to the complexity of the “tumor ecosystem” and the diversity of the stromal cell types that constitute the tumor microenvironment. Emerging data indicate that several factors, such as hypoxic stress, activate a plethora of resistance mechanisms, including autophagy, in tumor cells. Hypoxia-induced autophagy in the tumor microenvironment also activates several tumor escape mechanisms, which effectively counteract anti-tumor immune responses mediated by natural killer and cytotoxic T lymphocytes. Therefore, strategies aiming at targeting autophagy in cancer cells in combination with other therapeutic strategies have inspired significant interest to overcome immunological tolerance and promote tumor regression. However, a number of obstacles still hamper the application of autophagy inhibitors in clinics. First, the lack of selectivity of the current pharmacological inhibitors of autophagy makes difficult to draw a clear statement about its effective contribution in cancer. Second, autophagy has been also described as an important mechanism in tumor cells involved in presentation of antigens to T cells. Third, there is a circumstantial evidence that autophagy activation in some innate immune cells may support the maturation of these cells, and it is required for their anti-tumor activity. In this review, we will address these aspects and discuss our current knowledge on the benefits and the drawbacks of targeting autophagy in the context of anti-tumor immunity. We believe that it is important to resolve these issues to predict the use of autophagy inhibitors in combination with immunotherapies in clinical settings.

## Introduction

While initially considered as a disease of cells with deregulated gene expression, cancer progression is now considered to be largely influenced by the tumor microenvironment. It is now well established that factors in the tumor microenvironment play a key role in cancer progression, metastasis, and resistance to the therapies (1). In addition to malignant cells, the tumor microenvironment contains different subsets of immune cells, fibroblasts and cancer-associated fibroblasts, tumor vasculature and lymphatics, as well as pericytes and sometimes adipocytes (2). Effector immune cells infiltrating tumors, notably T lymphocytes and natural killer (NK) cells mediating adaptive and innate immunity, respectively, are basically the major immune cells able to kill cancer cells in the tumor microenvironment (3). Although these immune effectors are recruited to the tumor site, they are exhausted and their anti-tumor functions are often downregulated in response to microenvironmental factor such as hypoxia.

It is now widely accepted that the oxygen consumption of solid tumors is increased due to the tumor volume and elevation of the respiratory activity of different cell populations within a tumor. The increase in the oxygen consumption leads to the establishment of hypoxic tumor microenvironment. The hypoxic tumor microenvironment is a characteristic feature of locally advanced solid tumors and a major hallmark that contributes to tumor resistance to several therapies including chemotherapy, radiotherapy, and immunotherapy (4). While mounting experimental evidences highlight the role of hypoxia at primary tumors, the role of hypoxia in the metastatic dissemination and at the metastatic niches is only being unraveled. Indeed, hypoxia signaling pathway is involved in multiple steps of the metastatic cascade, including local invasion and migration, intravasation and extravasation, establishment of the pre-metastatic niche, and survival and growth at the distant site. The role of hypoxia in metastasis control is reviewed in many excellent reviews (5–7).

Hypoxia within the tumor is characterized by a condition where the pressure of oxygen is lower than 5–10 mm Hg. Such condition results from an insufficient and/or inadequate oxygen supply to the tumor bed. In normal tissues, the oxygen pressure is basically higher than that in the corresponding tumors. The oxygen pressure within the tumor likely depends on the initial oxygenation of the tissue as well as the heterogeneity and the size of the tumor. Table 1 shows the percentage of oxygen (reported as a median) in some healthy organs or tissues and their corresponding tumors. Adapted from Ref. (8).

**Table 1:** The median percentage of O_2_ in some organs and in their corresponding tumors.

Tissue/organ	Median % O_2_	Corresponding cancer	Median % O_2_
Brain	4.6	Brain tumor	1.7
Breast	8.5	Breast cancer	1.5
Kidney cortex	9.5	Renal cancer	1.3
Liver	4.0–7.3	Liver cancer	0.8
Lung	5.6	Non-small cell lung cancer	2.2
Pancreas	7.5	Pancreatic tumor	0.3
Rectal mucosa	3.9	Rectal carcinoma	1.8

Hypoxia is not only resulted from decrease in O_2_ partial pressure in arterial blood, but also from pathological conditions, such as anemia (anemic hypoxia), which restrict the ability of blood vessel to carry O_2_. It can also be generated from dramatic decrease in tissue perfusion or defect of cells to use O_2_. The level of O_2_ in tissue is finely tuned by blood flow regulatory mechanism, which is adapted according the consumption level of O_2_ in the tissue. Therefore, hypoxia can be generated in a particular tissue or organ if the system regulating blood flow fails to meet the level of O_2_ demand, thus impacting the function this tissue or organ. It should be noted that the term hypoxia has been used in several publications in a somewhat careless manner. Indeed, the *in vitro* experimental conditions described in many papers were routinely conducted under atmospheric O_2_ levels ranging from 18 to 21% O_2_. However, physiological normoxia comprises between 1 and 13% O_2_. Therefore, interpreting results when performing research under varying O_2_ conditions require a comprehensive understanding of physiological parameters that define the appropriate *in vitro* model.

Hypoxia induces disorganized tumor microvasculature and such abnormal tumor vascular network often fails to rectify the oxygen deficit. While normal tissue is composed of mature and well-organized blood vessels, abnormal tumor vasculature is largely composed of immature vessels characterized by increased permeability, vessel diameter, vessel length, vessel density, tortuosity, and interstitial fluid pressure. Such characteristics of tumor vasculature compromise the delivery of chemotherapeutic drugs and nutrients (9). While the role of hypoxia in tumor resistance to chemotherapy and radiotherapy is currently well described (10), emerging evidence points to its involvement in tumor resistance to immunotherapy. Indeed, experimental and clinical evidence suggests that the hypoxic tumor microenvironment is responsible for the establishment of large number of mechanisms suppressing the anti-tumor immune functions [reviewed in Ref. (11)]. We have shown that the anti-tumor immune response is dramatically impaired under hypoxic stress (12–17). It has been reported that the tumor-killing function of immune cells present in the hypoxic tumor microenvironment is largely attenuated and the immune cells at the hypoxic area of tumors displayed an anergic phenotype induced by malignant cell-derived factors (18). In addition, immune cells in the tumor microenvironment not only fail to perform their anti-tumor effector functions, but also they are co-opted to promote tumor growth (19). Thus, a hypoxic tumor microenvironment not only contributes to chemotherapy and radiotherapy resistance, but also induces the evasion of tumor cells from immunosurveillance. The compelling evidence for the involvement of hypoxia in tumor resistance to anti-cancer therapies makes it a high priority target for cancer therapy. Several preclinical and clinical trials have been initiated using hypoxia-activated prodrugs that target hypoxic tumor compartments or hypoxic bone marrow niches. However, despite compelling evidence highlighting the role of hypoxia in therapy resistance, several hypoxia-activated prodrugs failed to show efficacy in clinical trials (20). Such failure could be attributed to the lack of predictive biomarkers for hypoxia-activated prodrugs and to some technical challenges of assaying such drugs in appropriate clinical settings (20).

## Hypoxia Inducible Factor-1α (HIF-1α) is the Major Hypoxia Sensor

Hypoxic is sensed to a large extent by the HIF-1α. Briefly, the structure of HIF-1α composed of two oxygen-dependent degradation domains (ODDD) at the N-terminal (N-ODDD) and the C-terminal (C-ODDD) parts. In addition, HIF-1α displayed two transactivation domains (TADs), one N-terminal, which overlaps with the C-ODDD, and another C-terminal (21). HIF-1α is constantly synthesized in an O_2_-independent manner under normoxia, however, it is rapidly degraded by the ubiquitin proteasome system (UPS) in O_2_-dependent mechanism (22). Thus, under hypoxic stress, the decrease in the O_2_ pressure prevents the degradation of HIF-1α leading to its accumulation in the cytoplasm. It should be noted that, under normoxic conditions, the half-life of HIF-1α is very short, which is less than 5 min (23). The degradation of HIF-1α under normoxic conditions is related to its ability to be hydroxylated on proline residue 402 and/or 564 in the ODDD by prolyl hydroxylase domain protein 2 (PHD2) and its subsequent binding to the von Hippel–Lindau tumor suppressor protein (pVHL). pVHL is a component of an E3 ubiquitin-protein ligase complex that targets HIF-1α for proteolysis by the ubiquitin proteasome pathway (24).

Three prolyl hydroxylase domain (PHD) enzymes (PHD-1, PHD-2, and PHD-3) regulating HIF-1α proteasomal degradation have been identified (25, 26). Under hypoxia, the low O_2_ level inhibits the activity of PHD2, and HIF-1α is no longer hydro-xylated and its proteasomal degradation event is blocked (26). Therefore, HIF-1α is accumulated in the cytoplasm and then translocation to the nucleus. In the nucleus, HIF-1α dimerizes with HIF-1β and the HIF-1α/HIF-1β heteromer binds to the hypoxia responsive element in target genes before recruiting coactivators and inducing the transcription of several downstream target genes (27). More than 800 genes involved in several pathways and biological processes are reported to be transcriptionally activated by HIF-1α (21) since they contain in their promoter the core sequence 5′-[A/G]CGT-3′, which in most cases is ACGTG (28). Two other isoforms of HIFs family HIF-2α and HIF-3α have been identified; but only HIF-2α is stabilized by oxygen-dependent hydroxylation similar to HIF-1α (29). HIF-1α and HIF-2α share similar structure of their DNA binding and dimerization domains but differ in their TADs (30). HIF-3α functions as an inhibitor of HIF-1α and HIF-2α.

## Autophagy Activation by Hypoxic Stress in the Tumor Microenvironment

Macroautophagy (hereafter referred as autophagy) is an evolutionarily conserved cellular catabolic process responsible for the degradation of damaged proteins and organelles to produce alternative energy source necessary for maintaining cell homeostasis and viability. Although autophagy is executed at basal level in all cells, it is frequently increased in established tumors (31).

Basically, autophagy process contains three major steps: (i) the induction and phagophore formation; (ii) phagophore elongation and autophagosome formation; and (iii) fusion, degradation, and recycling. Briefly, the first step is initiated by a nucleation step or the formation of phagophore that involves two protein complexes: the class-III PI3K/Vps34, Atg6/Beclin1, and Atg14 and Vps15/p150 complex and the serine/threonine kinase Atg1/ULK1, which is a positive regulator of autophagosome formation. The maturation of the phagophore requires several autophagy-related proteins (ATG). During this step, portions of the cytoplasm are engulfed and the microtubule-associated protein 1 light chain 3 (LC3)-I is lipidated to LC3-II. During the maturation, the phagophore is closed by the action of LC3-II and BECN1 proteins, and this step is required for the formation of autophagosome. Materials intended to be degraded are finally sequestered in the autophagic vacuole that will be fused with lysosomes and subjected to degradation by lysosomal hydrolases (32).

Several studies reported that advanced tumors could be addicted to autophagy to maintain their energy balance (33, 34). Indeed, in cancer patients’ high autophagic index is correlated with less responsive to cancer therapy and worse survival compared with those with a low autophagic index (35). Therefore, autophagy has been recently considered as a major process in regulating the progression of hypoxic tumors.

Under hypoxia, autophagy is basically activated by three major pathways (36): low O_2_ pressure; unfolded protein response; and energy depletion. In this review, we will describe how autophagy is activated by low O_2_ level in tumors and summarize recent data describing how autophagy activation under low O_2_ pressure operating in tumor cells as a major resistance mechanism to anti-tumor immune response.

Hypoxia is a major characteristic of almost 50–60% of tumors (37), and that increased autophagy induces tumor cell survival (38). The stabilization of HIF-1α under hypoxia leads to its translocation to the nucleus. In the nucleus, HIF-1α induces the expression of downstream target genes, the BH3-only protein Bcl-2/adenovirus E1B 19 kDa-interacting protein 3 (BNIP3) and the related protein, BNIP3L (39). The upregulated expression of BNIP3 and BNIP3L dissociates Beclin1 from Bcl-2 and activates autophagy.

## Hypoxic Tumor Cells Activate Autophagy to Escape Cytotoxic T-Lymphocytes (CTL)-Mediated Killing

Several mechanisms have been described to induce hypoxic tumor cell escape from CTL-mediated killing. Bellow, we will briefly describe those involving autophagy activations.

### Hypoxia-Induced Autophagy Regulates Phospho-Signal Transducer and Activator of Transcription 3 (STAT3) Degradation

Signal transducer and activator of transcription 3 is a transcription factor that can be activated through phosphorylation by cytokine and growth factor signaling pathways including interleukin (IL)-6 (40), epidermal growth factor, and vascular endothelial growth factor (41). Following phosphorylation, STAT3 promotes tumor cell survival, proliferation, angiogenesis/metastasis, and immune escape (42–44). It has been reported that the immune escape properties of phospho-STAT3 relies on its ability to induce several genes responsible for immunosuppression (45–48). We have previously reported for the first time that hypoxic lung carcinoma cells can evade CTL-mediated killing by activating autophagy and that targeting autophagy by silencing ATG5, and Beclin1 was sufficient to restore their CTL-mediated killing (16, 49). We provided evidence that targeting autophagy in hypoxic cancer cells led to the accumulation of the adaptor protein sequestosome1 (SQSTM1/p62). Accumulated SQSTM1/p62 bound selectively to pSTAT3 and induced its selective degradation by the UPS. These data highlight targeting autophagy as a valuable strategy to improve CTL-mediated killing of hypoxic cancer cells. This statement was further supported by *in vivo* data using hydroxychloroquine (HCQ) as autophagy inhibitor in B16-F10 tumor-bearing mice (16). Thus, the effect of HCQ on the tumor growth of B16-F10 melanoma was assessed alone or in combination with a tyrosinase-related protein-2 (TRP2) peptide-based vaccination strategy. A synergistic effect on the inhibition of tumor growth was observed by combining HCQ with TRP2 vaccination, indicating that targeting autophagy represents an innovative strategy to improve the anti-tumor effect of TRP2-based vaccine.

### Hypoxia-Induced NANOG Expression Activates Autophagy by Regulating BNIP3L

In addition to the mechanism described above, other studies showed that hypoxia impaired CTL-mediated lysis by transcriptionally upregulating the stem cell self-renewal transcription factor NANOG (50, 51). It has been reported that targeting NANOG in hypoxic cells restored CTL-mediated tumor cell killing. In this regards, a link between NANOG expression and the phosphorylation of STAT3 has been proposed, since NANOG depletion results in the inhibition of STAT3 phosphorylation and its nuclear translocation. More recently, a direct regulation of autophagy inducer gene BNIP3L by NANOG has been reported by chromatin immunoprecipitation and luciferase reporter assays showing that NANOG binds directly to the enhancer sequence of BNIP3L and activates its transcription. These data strongly argue that the pluripotency factor NANOG and autophagy cooperate to induce resistance to CTL under hypoxia (52).

## Hypoxia-Induced Autophagy Leads to Tumor Cells Escape from NK-Mediated Killing

Similar to CTL, NK cells of the innate immune system able to recognize and kill tumor cells (53). The recognition and the killing of tumor cells by NK depend on the balance between the expression of activating and inhibitory receptors on the surface of NK cells and their corresponding ligands on the surface of tumor cells (54). Similar to CTL, NK cells kill their target following the establishment of immunological synapse (55) and the secretion of cytotoxic granules containing perforin and granzymes. In tumor cells, the secreted granules induce cell death by apoptosis (56). NK cells are also able to kill their target by tumor necrosis factor superfamily dependent mechanism (57). Below, we will briefly describe the major autophagy-related mechanisms responsible from tumor escape form NK-mediated killing.

### Hypoxia-Induced Autophagy in Tumor Cells Degrades NK-Derived Granzyme B

**Figure 1:**
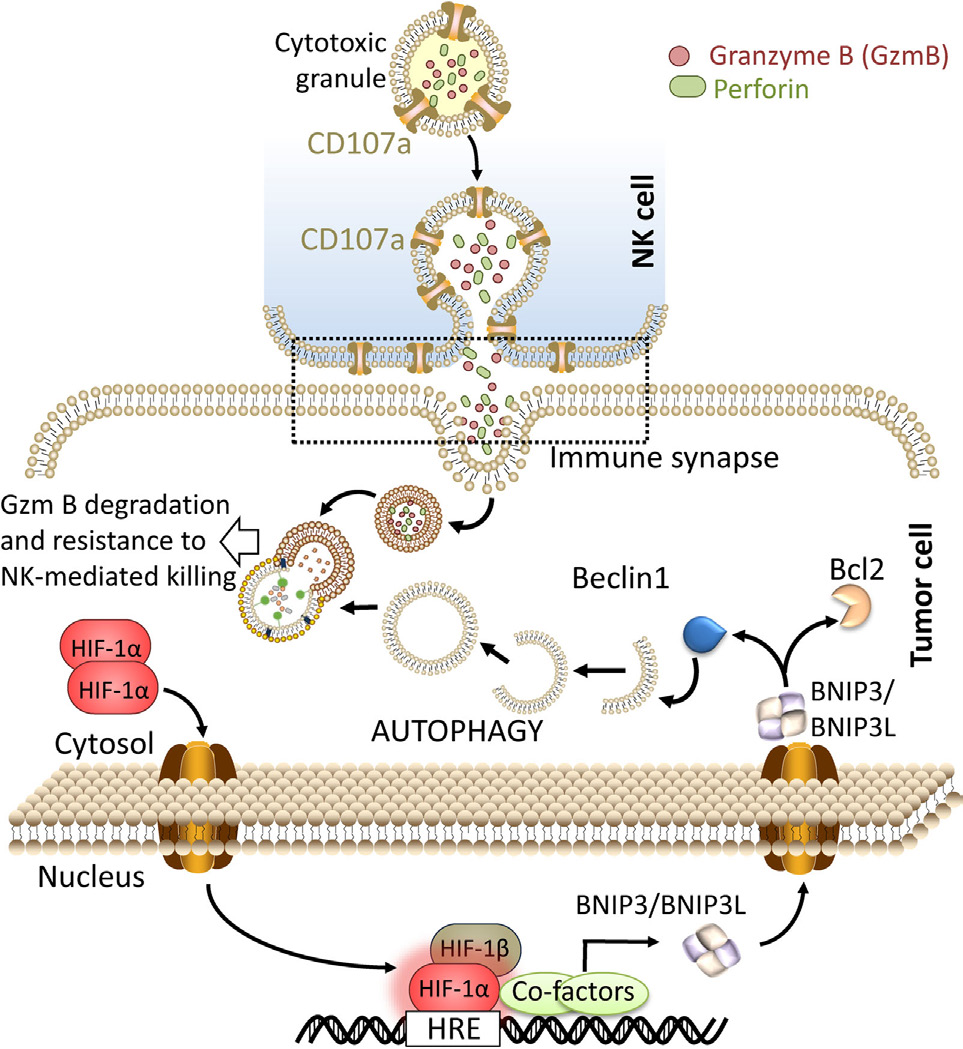
Targeting autophagy in hypoxic tumor cells restores natural killer (NK)-mediated tumor cell killing by preventing the degradation of granzyme B. The recognition of tumor cells by NK leads to the release of cytolytic granules containing perforin and granzyme B from NK cells. These cytotoxic granules enter to the tumor cells through endocytosis and traffic to enlarged endosomes called “gigantosomes.” Following the formation of pores in the “gigantosome” membrane, granzyme B is released in the cytoplasm and initiates cell death. Under hypoxia, excessive autophagy leads to the fusion of “gigantosomes” with autophagosomes and the subsequent degradation of granzyme B. Degraded granzyme B is no longer able to induce tumor cell death, therefore, targeting autophagy prevents the degradation of granzyme B and restores NK-mediated lysis.

We have reported that autophagy activation in tumor cells impaired NK-mediated killing by selective degradation of NK-derived granzyme B in the lysosome compartment. Using GFP granzyme B-expressing NK cells, we provided evidence that the level of granzyme B is significantly lower in hypoxic tumor cells compared with normoxic tumor cells. Targeting autophagy by knocking down Beclin1 in hypoxic tumor cells was sufficient to rescue the granzyme B level in hypoxic cells and restore NK-mediated lysis (12, 58, 59). These data clearly suggest that during its intracellular trafficking in hypoxic tumor cells, granzyme B is exposed to a high risk of being targeted to autophagosomes and subsequently to the lysosome compartment to be degraded (Figure 1). While autophagy has long been considered as a process of non-selective bulk degradation, new evidence suggested that it can be a selective degradation process under stress conditions. The selectivity of autophagy to degrade specific proteins depends on several cargo protein including SQSTM1/p62. In keeping with this, no data are available so far describing whether granzyme B is selectively degraded by autophagy or it is just an “innocent victim” subjected to non-specific degradation under hypoxia in tumor cells.

### Targeting Autophagy Induces a Massive Infiltration of NK Cells into the Tumor Bed

**Figure 2:**
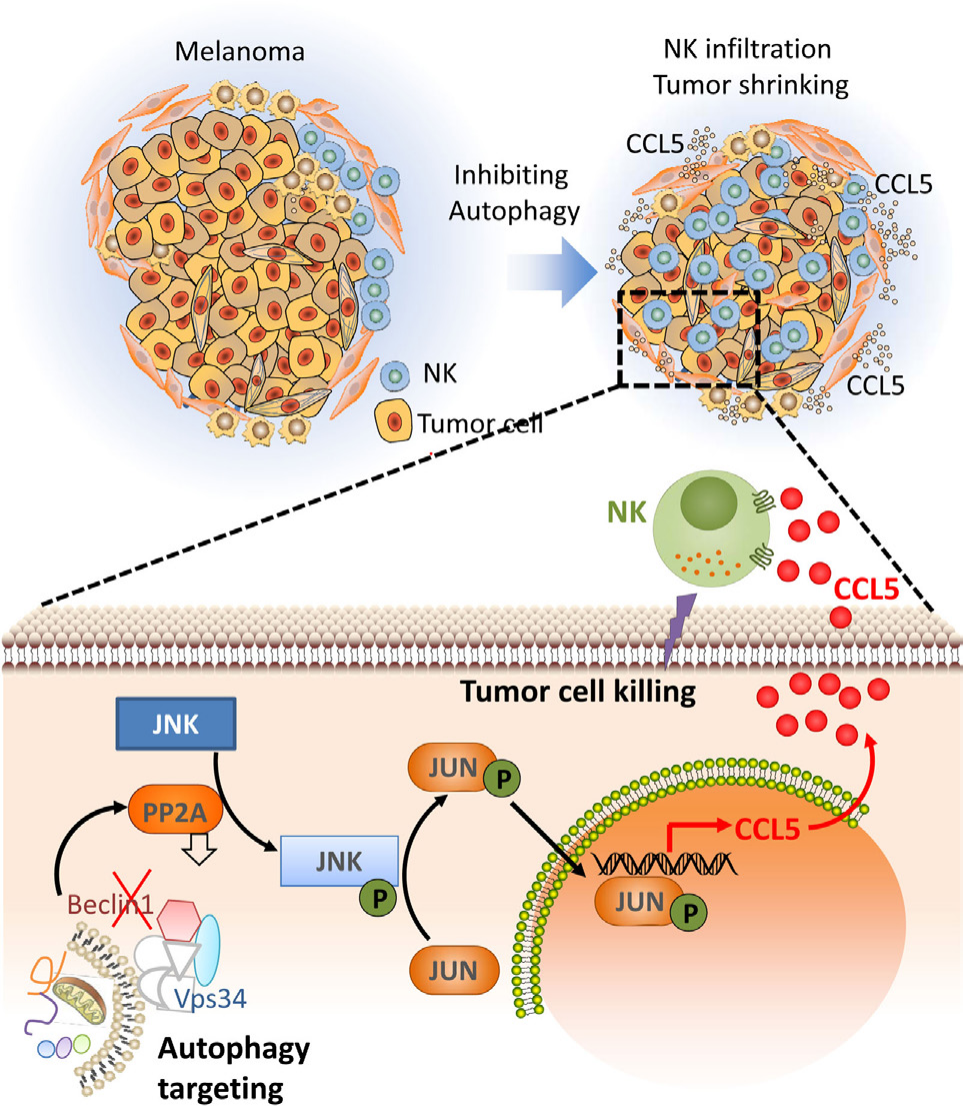
Targeting autophagy leads to tumor regression by inducing the infiltration of natural killer (NK) cells into the tumor bed. Targeting autophagy in tumor cells induces the expression of CCL5 cytokine. Through paracrine mechanism, CCL5 binds its receptors expressed on the surface of NK cells and induces the recruitment of functional NK cells to the tumor bed. Functional NK cells recruited to the tumor kill cancer cells leading to tumor regression. The lower part describes the molecular mechanism underlying the expression of the chemotactic cytokine CCL5. Briefly, targeting Beclin1 leads to a decrease in the activity of the protein phosphatase 2A by a mechanism not yet understood. Such a decrease enhances the phosphorylation of JNK that subsequently phosphorylates c-JUN. Phosphorylated c-JUN binds to the promoter of Ccl5 and induces its transcription. CCL5 released by Beclin1-defective tumor cells binds to CCL5 receptor on the surface of NK cells, and induces their infiltration. Functional NK cells recruited to the tumor site kill cancer cells and thereby reduce the tumor volume.

Based on our data showing that targeting autophagy restores tumor cell susceptibility to NK-mediated lysis *in vitro*, we investigated whether blocking autophagy reduces tumor growth in an NK-dependent manner. We used BALB/c and C57BL/6 mice transplanted with syngeneic murine 4T1 breast adenocarcinoma and B16-F10 melanoma tumor cells, respectively. We first showed that the tumor growth of these two syngeneic mouse models is primarily controlled by NK cells as the depletion of host NK cells significantly increases tumor growth. We next assessed the impact of targeting Beclin1 on the tumor growth. Our data showed that targeting Beclin1 resulted in a significant decrease in the tumor growth presumably as a consequence of potentiation of tumor cell killing by NK cells. The decrease in the tumor growth was no longer observed when NK cells were depleted. In keeping with this, we showed a massive infiltration of NK cells into Beclin1-defective compared with control B16-F10 tumors. Mechanistically, we showed that the infiltration of NK cells is related to the ability of Beclin1-defective tumor cells to overexpress CCL5 cytokine responsible for the trafficking of NK cells to the tumor (Figure 2). The infiltration of NK cells was completely abrogated when CCL5 was silenced in Beclin1-defective tumor. Furthermore, we showed that the overexpression of CCL5 involved the activation of the transcription factor c-Jun by JNK (60).

## Targeting Autophagy in the Context of Cancer Therapy: Friend or Foe?

Several lines of evidence supports the concept that autophagy activation is associated with cancer cell resistance to chemotherapy (61, 62), radiotherapy (63, 64) and immunotherapy (12, 16, 58) either by supporting cell metabolism directly (65) or through the impairment of cell death pathway (66). Therefore, several preclinical and clinical studies have been undertaken to develop drugs able to inhibit autophagy (67). Basically, pharmacological inhibitors of autophagy pathway can be classified into three classes: (i) inhibitors of the initiation step of autophagy; (ii) inhibitors of the nucleation of phagophore; and (iii) inhibitors of the fusion of autophagosomes with lysosomes [reviewed in Ref. (67, 68)]. In this review, we will not describe all drugs inhibiting each step of autophagy but briefly describe the action of those displaying potent anti-tumor activities.

Chloroquine (CQ) has been approved for decades in the treatment of malaria and arthritis, and currently used as autophagy inhibitors. CQ blocks the last step of autophagy process before the fusion of autophagosomes with lysosomes (69). Therefore, several clinical trials are currently evaluating CQ or its derivative HCQ alone or in combination with chemotherapy or radiotherapy in patients with several types of cancers (70). Briefly, a significantly prolonged median survival of glioblastoma (GBM) patients (33 months compared with 11 months) was observed using CQ combination with temozolomide and radiotherapy (40). The combination of CQ with radiotherapy also reported in a pilot and phase II clinical trials to improve the survival of non-small cell lung carcinoma, squamous cell lung carcinoma, and breast and ovarian cancer patient with brain metastasis (71). Another phase I/II clinical trial using CQ in combination with radiotherapy in GBM showed no significant improvement in the survival (72) due to an inconsistent inhibition of autophagy between patients and dose-limiting toxicities that prevented the use of high CQ doses. In some trials, CQ was also used as monotherapy, notably in patients with metastatic pancreatic cancer, but no clinical benefit was observed. This failure to provide clinical benefit could be related to inconsistent autophagy inhibition was reported (72) and the limited potential for CQ as single agent to improve end-stage disease outcomes. However, in PDX preclinical model, the single treatment with HCQ was effective (73). The combination of HCQ and gemcitabine in preoperating setting of patients with pancreatic adenocarcinoma induced a decrease in the serum tumor marker cancer antigen 19-9 in 61% (74). In the context of cancer immunotherapy, the effect of CQ has been evaluated in combination with high-dose interleukin-2 (HDIL-2) in preclinical murine hepatic metastasis model. Combining CQ with HDIL-2 enhanced IL-2 immunotherapeutic efficacy and limit toxicity by increasing long-term survival, decreased toxicity associated with vascular leakage, and enhanced immune cell proliferation and infiltration in the liver and spleen (75).

Based on studies described above, it appears that the clinical response to autophagy inhibitors varied widely. The major difficulties were the identification of appropriate pharmaco-dynamic biomarkers to evaluate the change in autophagy (70). Therefore, none of them formally confirmed that inhibiting autophagy in cancer cells provides therapeutic benefits to cancer patients (76). It remains to be defined whether the lack of therapeutic benefits is related to the lack of the specificity of CQ to inhibit autophagy. Indeed, it should be highlighted that CQ and HCQ are non-selective autophagy inhibitors since they lead to the reduction of nutrient scavenging (77, 78). They could also alter tumor pH, thus affecting other drugs bioavailability when combined with conventional cytotoxic chemotherapies (79). Currently, there is a major interest in developing selective new drugs inhibiting autophagy as an important survival mechanism of tumors.

Lys05 is dimeric form of CQ displaying more potent autophagy inhibitor than CQ, which displays more potent accumulation properties in the lysosome. Lys05 is, therefore, a new lysosomal autophagy inhibitor with a strong potential to be developed into a drug for cancer. It has been reported that Lys05 is a potent anti-tumor drug *in vitro* and in several preclinical mouse model. The potent autophagy inhibition property of Lys05 relied to the bivalent aminoquinoline rings, C7-Chlorine, and a short triamine linker. Since Lys05 is a potent inhibitor of autophagy it can be used at low doses, which are well tolerated and associated with strong anti-tumor activity (80).

Another druggable autophagy target proteins have been recently proposed, which include Beclin-1 and Vps34 (or PI3K class-III) (81). Both of them are involved in the early step of autophagy initiation (82, 83). SAR405 is a kinase inhibitor of Vps18 and Vps34. The inhibition of Vps34 leads to an impairment in the lysosomal function, thus affecting vesicle trafficking between late endosome and the lysosome. The Vps34i (SAR405) has been developed following chemical optimization with highly potent and selective inhibitor of vesicle trafficking from late endosomes to lysosomes. SAR405 inhibits also starvation- and mTOR-dependent induction of autophagy (84, 85).

Another autophagy druggable protein is the serine/threonine kinase ULK1/Atg1 involved in the core autophagy pathway. Cell-based screen allowed identification of a potent ULK1 small molecule inhibitor SBI-0206965. This drug is highly selective ULK1 kinase inhibitor *in vitro* and suppressed ULK1-mediated phosphorylation events in cells. The anti-tumor activity of SBI-0206965 has been proved *in vivo*, thus providing a strong rationale for it use in the clinic (86). NSC185058 has been identified as an effective inhibitor of ATG4B activity. NSC185058 showed a negative impact on the development of Saos-2 osteosarcoma tumors *in vivo* (87). Inhibition of ATG4B using NSC185058 was reported to reduce autophagy and tumorigenicity of GBM cells and to improve the impact of radiotherapy on GBM growth in mice (88). These results suggest that ATG4B is another suitable anti-autophagy target and a promising therapeutic target to treat osteosarcoma.

Beside its role in supporting tumor growth and resistance to therapies, preclinical results suggest that intact autophagic responses in cancer cells are dispensable for the initiation of an appropriate danger signaling and thus for the initiation appropriate anti-cancer immune responses in syngeneic tumor models treated with immunogenic chemotherapy or radiotherapy (89, 90). Indeed, by contrast to autophagy-defective tumors, autophagy-competent tumors attracted dendritic cells and T lymphocytes into the tumor bed. Inhibiting autophagy impaired the immunogenic release of adenosine triphosphate (ATP) from dying tumor cells and subsequently blocked the ATP-dependent recruitment of immune cells (89).

In addition its impact on tumor cells, it has been observed that autophagy actively participates in the intracellular antigen processing for major histocompatibility complex (MHC) class-II and I presentation as well as in extracellular antigen processing for MHC class-II presentation. It has been also reported that autophagy is involved in the cross-presentation of antigens for MHC class-I presentation and in MHC class-I internalization [reviewed in Ref. (91)]. In keeping with this, it appears that the autophagic machinery plays an important role in many aspects of the antigen presentation and therefore raises the question about the net outcome of inhibiting autophagy on the adaptive immunity.

In addition to the role of autophagy in antigens processing, autophagy plays a functional role in different immune cell type. Briefly, in macrophages autophagy plays a crucial role in macrophage homeostasis by different mechanisms [reviewed in Ref. (59)]. The autophagic activity is increased in DCs compared with other cell types. Such autophagic activity is related to intensive processing of extra- and intra-cellular antigens for the MHC class-I and -II presentation (92).

The role of autophagy in T cells was also addressed. In the context of naive T cells, it has been reported that tumor-derived metabolite lactate selectively inhibits FAK family–interacting protein of 200 kDa (FIP200; also known as RB1CC1) in naive T cell leading to autophagy deficiency, apoptosis and poor anti-tumor immunity in ovarian cancer patients, and tumor-bearing mice (93).

In tumor cells, suppression of FIP200 suppresses the initiation and progression of mammary tumor breast cancer driven by the PyMT oncogene. In addition, FIP200 conditional knockout mice display elevated expression level of interferon (IFN)-responsive genes associated with increased infiltration of effector T cells in the tumor microenvironment triggered by the production of CXCL10 chemokine (94). In regulatory T (Treg) cells, autophagy plays a major role in their lineage stability and survival fitness. Specific ablation of autophagy-related genes Atg7 or Atg5 in Treg induces apoptosis and loss of Foxp3 transcription factor (95). In KRas^G12D^-driven lung cancer mouse model, it has been reported that ablation of Atg5 favors adenosinergic signaling *via* a HIF-1α pathway, as well as the infiltration of tumors by Tregs, thus influencing inflammatory and immunosurveillance mechanisms that can stimulate and control carcinogenesis, respectively (96). Pharmacological blocking of autophagy by CQ enhances IL-2 immunotherapeutic efficacy and limit toxicity. Combining CQ with IL-2 increases long-term survival, decreases toxicity associated with vascular leakage, and enhances immune cell proliferation and infiltration in the liver and spleen (75). These results support the use of autophagy inhibitors as a novel clinical strategy to enhance the efficacy of IL-2-based immunotherapy for cancer patients. Similarly, the ablation of autophagy-related gene GABARAP, inhibits the tumor formation incidence in mice and by enhancing the immune response through increased secretion of IL-1β, IL-6, IL-2, and IFN-γ from stimulated macrophages and lymphocytes (97).

Furthermore, autophagy seems to be an important mechanism for the development, maintenance, and survival of T lymphocytes (98–100). Moreover, the interaction of B cells with CD4+ T cells requires autophagy that promotes the presentation of antigens by MHC class-II molecules through a mechanism reminiscent to that described for DCs (101, 102).

## Concluding Remarks

Given the impressive impact of targeting autophagy on tumor immunity is the ultimate question that arises whether targeting autophagy would improve or impair the efficacy of cancer immunotherapy. Based on our current knowledge available so far, it is difficult to draw a clear statement about this question. In this review, we provided some clues to argue that blocking autophagy for therapeutic purposes requires careful consideration. Although targeting autophagy appears to improve the anti-tumor immune response, it should be highlighted that such strategies must consider the potential negative or positive impact on immune cells. Therefore, it is important to evaluate the net outcome of targeting autophagy in the context of the TME rather than analyzing the impact of targeting autophagy at the cellular level. Moreover, considering this complex role of autophagy in the tumor microenvironment it is still difficult to draw a clear statement whether, when, and how autophagy has to blocked or enhanced for the benefit of cancer patients.

## Author Contributions

BJ, GB, and SC wrote the manuscript. BJ designed figures.

## Conflict of Interest Statement

The authors declare that the research was conducted in the absence of any commercial or financial relationships that could be construed as a potential conflict of interest.
